# *CACNA1A* Mutations Associated With Epilepsies and Their Molecular Sub-Regional Implications

**DOI:** 10.3389/fnmol.2022.860662

**Published:** 2022-05-04

**Authors:** Xue-Lian Li, Zong-Jun Li, Xiao-Yu Liang, De-Tian Liu, Mi Jiang, Liang-Di Gao, Huan Li, Xue-Qing Tang, Yi-Wu Shi, Bing-Mei Li, Na He, Bin Li, Wen-Jun Bian, Yong-Hong Yi, Chuan-Fang Cheng, Jie Wang

**Affiliations:** ^1^Key Laboratory of Neurogenetics and Channelopathies of the Ministry of Education of China, Department of Neurology, Institute of Neuroscience, The Second Affiliated Hospital of Guangzhou Medical University, Guangzhou, China; ^2^Department of Neurology, The Affiliated Yuebei People’s Hospital of Shantou University Medical College, Shaoguan, China; ^3^Department of Cardiology, The Second Affiliated Hospital of Guangzhou Medical University, Guangzhou Medical University, Guangzhou, China

**Keywords:** *CACNA1A*, partial epilepsy, childhood absence epilepsy, genotype-phenotype correlation, molecular sub-regional implication

## Abstract

**Purpose:**

Previously, mutations in the voltage-gated calcium channel subunit alpha1 A (*CACNA1A*) gene have been reported to be associated with paroxysmal disorders, typically as episodic ataxia type 2. To determine the relationship between *CACNA1A* and epilepsies and the role of molecular sub-regional on the phenotypic heterogeneity.

**Methods:**

Trio-based whole-exome sequencing was performed in 318 cases with partial epilepsy and 150 cases with generalized epilepsy. We then reviewed all previously reported *CACNA1A* mutations and analyzed the genotype-phenotype correlations with molecular sub-regional implications.

**Results:**

We identified 12 *CACNA1A* mutations in ten unrelated cases of epilepsy, including four *de novo* null mutations (c.2963_2964insG/p.Gly989Argfs*78, c.3089 + 1G > A, c.4755 + 1G > T, and c.6340-1G > A), four *de novo* missense mutations (c.203G > T/p.Arg68Leu, c.3965G > A/p.Gly1322Glu, c.5032C > T/p.Arg1678Cys, and c.5393C > T/p.Ser1798Leu), and two pairs of compound heterozygous missense mutations (c.4891A > G/p.Ile1631Val& c.5978C > T/p.Pro1993Leu and c.3233C > T/p.Ser1078Leu&c.6061G > A/p.Glu2021Lys). The eight *de novo* mutations were evaluated as pathogenic or likely pathogenic mutations according to the criteria of American College of Medical Genetics and Genomics (ACMG). The frequencies of the compound heterozygous *CACNA1A* mutations identified in this cohort were significantly higher than that in the controls of East Asian and all populations (*P* = 7.30 × 10^–4^, *P* = 2.53 × 10^–4^). All of the ten cases were ultimately seizure-free after antiepileptic treatment, although frequent epileptic seizures were observed in four cases. Further analysis revealed that episodic ataxia type 2 (EA2) had a tendency of higher frequency of null mutations than epilepsies. The missense mutations in severe epileptic phenotypes were more frequently located in the pore region than those in milder epileptic phenotypes (*P* = 1.67 × 10^–4^); *de novo* mutations in the epilepsy with intellectual disability (ID) had a higher percentage than those in the epilepsy without ID (*P* = 1.92 × 10^–3^).

**Conclusion:**

This study suggested that *CACNA1A* mutations were potentially associated with pure epilepsy and the spectrum of epileptic phenotypes potentially ranged from the mild form of epilepsies such as absence epilepsy or partial epilepsy, to the severe form of developmental epileptic encephalopathy. The clinical phenotypes variability is potentially associated with the molecular sub-regional of the mutations.

## Introduction

The voltage-gated calcium channel subunit alpha1 A gene (*CACNA1A*; MIM: 601011), located at chromosome locus19p13.13 and covering approximately 417 kb of genomic DNA with 47 exons, is predominantly expressed in the central nervous system (Kramer et al., 1995; Teh et al., 1995). It encodes the voltage-dependent P/Q-type calcium channel subunit alpha-1A (Cav2.1) that primarily distributed in nucleus, plasma membrane, neuronal cell body, and synapse (Ophoff et al., 1996). Cav2.1 is the alpha-1A subunit of the voltage-gated calcium channel (VGCC) that mediates the entry of calcium ions into excitable cells and are also involved in a variety of calcium-dependent processes, including muscle contraction, hormone or neurotransmitter release, and gene expression (Diriong et al., 1995). As a component of VGCC, Cav2.1 forms the pore region of the calcium channel and directs the channel activity (Tuluc et al., 2021).

Mutations in *CACNA1A* gene have been demonstrated to be associated with a wide range of paroxysmal diseases, such as episodic ataxia type 2 (EA2; MIM: 108500), familial hemiplegic migraine 1 (FHM1; MIM: 141500), spinocerebellar ataxia 6 (SCA6; MIM: 183086), and developmental epileptic encephalopathy 42 (DEE42; MIM: 617106). Cases with EA2 or FHM1 may be complicated by epilepsy or seizures that were generally mild phenotypes (Imbrici et al., 2004; Du et al., 2017). *CACNA1A* mutations have also been occasionally identified in patients with mild form of epilepsy, including absence epilepsy, juvenile myoclonic epilepsy, and idiopathic epilepsy (Klassen et al., 2011; Helbig et al., 2016; Lee et al., 2018). These findings suggest *CACNA1A* is possibly related to human epilepsies. However, the relationship between *CACNA1A* and epilepsies has not been defined and it is unknown the genotype-phenotype correlation in the spectrum of *CACNA1A*-associated disorders.

In this study, we performed trio-based whole-exome sequencing (WES) in a cohort of patients with epilepsy. Twelve *CACNA1A* mutations were identified in ten unrelated cases with phenotypic heterogeneity. We further systematically reviewed all *CACNA1A* mutations and analyzed their molecular heterogeneity, aiming to clarify the mechanism underlying phenotypical variation and the role of molecular sub-regional effect.

## Materials and Methods

### Patients

The patients were recruited at the Epilepsy Center of the Second Affiliated Hospital of Guangzhou Medical University and the Affiliated Yuebei People’s Hospital of Shantou University Medical College. Clinical phenotypes of epileptic seizures and epilepsy syndromes were assessed following the criteria of the Commission on Classification and Terminology of the International League Against Epilepsy (ILAE) (1981, 1989, 2001, 2010, 2017). Partial epilepsy was used to denote cases with partial seizures and EEG features of idiopathic epilepsy including shift, bilateral or multiple focal discharge. Generalized epilepsy was diagnosed on the basis of typical generalized seizures, such as absence, myoclonic, atonic, and generalized tonic-clonic seizures, supported by the results of generalized discharges on EEG. Participants with acquired causes like brain malformation, infection or metabolic disorders were excluded. We collected the comprehensive clinical materials, including gender, current age, seizure onset age, seizure type and frequency, outcome, response to antiepileptic drugs (AEDs), family history, and results from general and neurological examinations. Brain CT or MRI scans were performed to detect abnormalities in brain structure. Long-term (24 h) video EEGs that included open-close eyes test, hyperventilation, intermittent photic stimulation and sleep recording, were performed and the results were double-reviewed by two qualified researchers. All individuals enrolled were unrelated ethnic Han Chinese with four Han Chinese grandparents, and were born to non-consanguineous Chinese parents. All of subjects were followed up for at least 1 year at epilepsy centers. A total of 468 cases were recruited, including 318 cases with partial epilepsy and 150 cases with generalized epilepsy. Additionally, we recruited 296 healthy Chinese volunteers as a normal control group as our previous report (Wang et al., 2018, 2020, 2021).

All procedures in this study involving human participants have been approved by the ethics committee of the Second Affiliated Hospital of Guangzhou Medical University. Written informed consents have been obtained from all participants or their parents/legal guardians in the case of child or those with intellectual disability.

### Whole-Exome Sequencing and Genetic Analysis

The genomic DNAs were extracted from the peripheral blood samples of the probands, their parents, and available family members using the FlexiGene DNA kit (Qiagen). Trio-based whole-exome sequencing was performed on an Illumina HiSeq 2000 sequencing platform as previously reported (Shi et al., 2019; Wang et al., 2021). To obtain high-quality reads, the massive parallel sequencing was performed with more than 125 times average depth and more than 98% coverage in the capture region of the chip. The original read data were aligned to the Genome Reference Consortium Human Genome build 37 (GRCh37) using Burrows-Wheeler alignment (BWA) with default parameters. Variant calling and quality filtration were conducted using the Genome Analysis Toolkit (DePristo et al., 2011).

To derive the whole candidate pathogenic variants in each trio, we adopted a case-by-case analytical approach as previously described (Zhou et al., 2018; Wang et al., 2021). Initially, we removed the common variants presenting a minor allele frequency ≥ 0.005 in the Genome Aggregation Database (gnomAD).^1^ We then prioritized potentially pathogenic variants, including frameshift, nonsense, canonical splice site, initiation codon, and missense variants predicted as being damaging *in silico* tools (VarCards).^2^ We screened *CACNA1A* mutations with origination of explainable for genetic diseases, including *de novo* mutation, mutation with segregations, and homozygous/compound heterozygous mutation. Additionally, I-Mutant 3.0 program was applied^3^ to predict the effect of *CACNA1A* missense variants on protein stability, which was indicated by free energy change (DDG). Negative DDG value means that the mutated protein possesses less stability and vice versa. Eventually, the pathogenicity of the identified *CACNA1A* mutations was evaluated by American College of Medical Genetics and Genomics (ACMG) scoring (Richards et al., 2015). Polymerase chain reaction and sanger sequencing was performed to validate the identified potential pathogenic variations by using ABI 3730 sequencing platform (Applied Biosystems, Foster City, CA, United States). All *CACNA1A* mutations identified in this study were annotated to reference transcript NM_001127222.

### Genotype-Phenotype Relationship

All *CACNA1A* mutations and related phenotypes were systematically retrieved from the professional edition of Human Gene Mutation Database (HGMD)^4^ and the PubMed database^5^ up to December 2021. To explore the relationship between genotype and phenotype, we divided the *CACNA1A* mutations into two categories, destructive (null) and missense mutations. Null mutations were those causing gross malformation of the gene/protein and leading to loss of function and haploinsufficiency (Richards et al., 2015; Liu et al., 2020), including truncating mutations (non-sense and frameshifting), splice site mutations (canonical ± 1 or 2), and mutations at initiation codon or with single/multi exon deletion. To facilitate analyzing the effect of molecular sub-regional on epileptic phenotypes, we collected the information on the distribution of *CACNA1A* missense mutations in various regions of the Cav2.1 channel. Considering that epileptic phenotype severity may be associated with mutation origin (*de novo* or inherited), we also collected the inheritance information of epilepsy associated *CACNA1A* mutations.

### Statistical Analysis

Statistical analyses were performed in GraphPad Prism version 8.00. A two-tailed Fisher’s exact test was used to compare the frequencies of null mutation, missense mutation, and *de novo* mutation between different phenotype groups. The recessive *CACNA1A* variants burden was also analyzed according to a recent research (Martin et al., 2018). The cutoff value for statistical significance is 0.05.

## Results

### Identification of Novel *CACNA1A* Mutations

**FIGURE 1:**
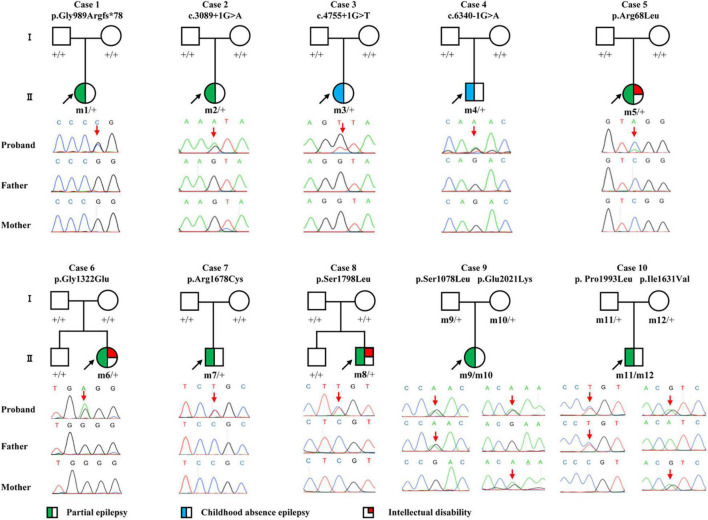
Pedigrees and DNA sequencing chromatograms of the cases with *CACNA1A* mutations. Individuals with heterozygous mutation are indicated by m/ +, those with compound heterozygous mutation are indicated by m/m, and those negative for mutation are indicated by +/ +. The probands are indicated by black arrows. The positions of the mutations are indicated by red arrows. The phenotype of each case is indicated by different symbols in the figure.

**FIGURE 2:**
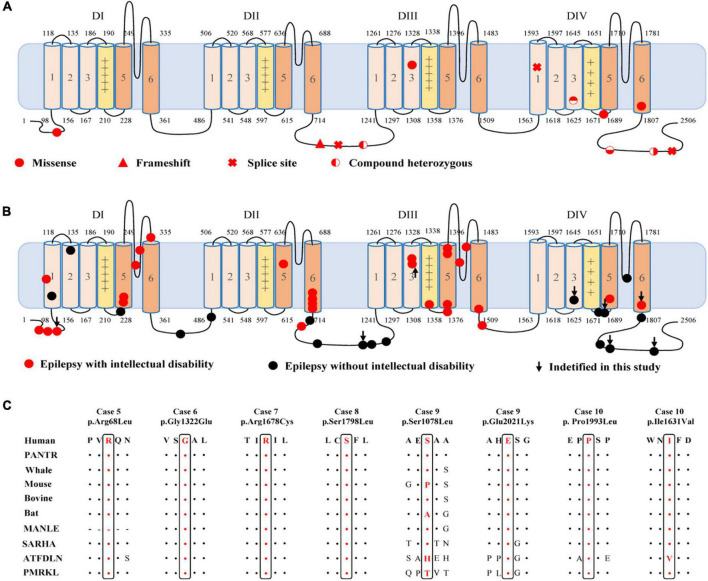
Location of the identified *CACNA1A* mutations in Cav2.1 and amino acid sequence alignment of the missense mutations. **(A)** Schematic illustration of the Cav2.1 protein and the location of the *CACNA1A* mutations identified in this study. **(B)** Schematic illustration of the Cav2.1 protein and the location of the epilepsy-related *CACNA1A* missense mutations. **(C)** Amino acid sequence alignment of the eight missense mutations with protein substitutions show that Arg68, Gly1322, Arg1678, Ser1798, Pro1993, and Glu2021 are highly conserved across species. Ser1078 and Ile1631 show a low degree of conservation.

Twelve *CACNA1A* mutations were identified in ten unrelated cases of epilepsy, including four null mutations (c.2963_2964insG/p.Gly989Argfs*78, c.3089 + 1G > A, c.4755 + 1G > T, and c.6340-1G > A) and eight missense mutations (c.203G > T/p.Arg68Leu, c.3233C > T/p.Ser1078Leu, c.3965G > A/p.Gly1322Glu, c.4891A > G/p.Ile1631Val, c.5032C > T/p.Arg1678Cys, c.5393C > T/p.Ser1798Leu, c.5978C > T/p.Pro1993Leu, and c.6061G > A/p.Glu2021Lys). The Ser1798Leu mutation has previously been described as a *de novo* mutation in a case of EA2 (Ohba et al., 2013), whereas the remained 11 mutations have not been reported and were novel findings. Four of the missense mutations (c.3233C > T/p.Ser1078Leu&c.6061G > A/p.Glu2021Lys and c.4891A > G/p.Ile1631Val&c.5978C > T/p.Pro1993Leu) constituted two pairs of compound heterozygous mutations; the remaining four missense mutations and four null mutations were *de novo* (Figures 1, 2 and Table 1). The eight *de novo* mutations were neither in gnomAD populations nor in our 296 normal control subjects and were evaluated as pathogenic or likely pathogenic mutations according to the criteria of ACMG (Table 2). The two pairs of the compound heterozygous mutations were absent in our 296 normal control subjects and present in gnomAD with an extremely low frequency (Table 2). When the recessive variants burden was analyzed, a statistically significant difference of the compound heterozygous *CACNA1A* mutations in this cohort was observed comparing the excepted number by chance in the controls of East Asian and all populations in the Exome Aggregation Consortium (*P* = 7.30 ×10^–4^, *P* = 2.53 × 10^–4^) (Martin et al., 2018).

**TABLE 1:** Clinical feature of the individuals with *CACNA1A* mutations.

Case	Mutation (NM_001127222)	Gender	Age	Onset age	Seizure course	Seizure-free duration	Effective AEDs	EEG	Brain imaging	Development	Diagnosis
Case 1	p.Gly989Argfs*78	Female	24 yr	3 yr	SPS, 1–2/mo and up to 2/wk for 9 yr	12 yr	VPA	Diffuse SW, irregular sharp and spike waves	Normal	Normal	PE
Case 2	c.3089 + 1G > A	Female	3 yr	1 yr	1–2/mo for 1 yr	1 yr	VPA	Bilateral occipital SSW	Normal	Normal	PE
Case 3	c.4755 + 1G > T	Female	9 yr	2 yr	FS twice at 2 yr, Ab, 10–20/d from 8 yr to 8.5 yr	0.5 yr	VPA	Ictal: 10 Ab; interictal: paroxysmal 3 HZ SSW.	Normal	Normal	CAE
Case 4	c.6340-1G > A	Male	10 yr	6 yr	Ab, 5–6/d for 2 yr	2 yr	VPA	Paroxysmal generalized 3 Hz SSW	Normal	Normal	CAE
Case 5	p.Arg68Leu	Female	21 yr	11 yr	sGTCS, 1–2/mo for 6 yr	4 yr	VPA, LTG	Right frontal and temporal spikes and FSW	Normal	ID	PE, ID
Case 6	p.Gly1322Glu	Female	4 yr	3 mo	sGTCS and CPS, 3–4/d for 1.5 yr	2 yr	VPA, LTG	Left parietal and temporal sharp waves and FSW	Normal	ID	PE, ID
Case 7	p.Arg1678Cys	Male	13 yr	10 yr	SPS, 1–2/mo for 2 yr	1 yr	OXC	Bilateral occipital sharp waves	Normal	Normal	PE
Case 8	p.Ser1798Leu	Male	5 yr	1.5 yr	sGTCS and CPS, 1–2/mo for 2.5 yr	1 yr	VPA	Bilateral occipital spikes and FSW	Normal	ID	PE, ID
Case 9	p.Ser1078Leu p.Glu2021Lys	Female	7 yr	4 yr	FS once at 4 yr, sGTCS and CPS, 1–4/wk for 2 yr	1 yr	VPA, OXC	Bilateral frontal and central sharp waves	Normal	Normal	PE
Case 10	p.Ile1631Val p.Pro1993Leu	Male	10 yr	1 yr	FS 1–2/yr for 4 yr, CPS once at 7 yr	3 yr	LEV	Left parietal and temporal spikes	Normal	Normal	PE

*Ab, absence; AEDs, antiepileptic drugs; CAE, childhood absence epilepsy; CPS, complex partial seizure; d, days; EEG, electroencephalogram; FS, febrile seizure; FSW, focal sharp and slow wave; ID, intellectual disability; LEV, levetiracetam; LTG, lamotrigine; mo, months; OXC, oxcarbazepine; PE, partial epilepsy; sGTCS, secondary generalized tonic-clonic seizure; SPS, simple partial seizure; SSW, spike and slow wave; SW, slow waves; VPA, valproate; wk, weeks; yr, years.*

**TABLE 2:** Genetic characteristic and ACMG scoring of the *CACNA1A* mutations.

Case no.	Mutation	Inheritance	MAF	MAF-EAS	SIFT^a^	PP2_Var^a^	MutationTaster^a^	M_CAP^a^	DDG (kcal/mol)	ACMG scoring	ACMG pathogenicity
Case 1	p.Gly989Argfs*78	*De novo*	–	–	–	–	–	–	–	PVS1 + PS2 + PM2	Pathogenic
Case 2	c.3089 + 1G > A	*De novo*	–	–	–	–	–	–	–	PVS1 + PS2 + PM2	Pathogenic
Case 3	c.4755 + 1G > T	*De novo*	–	–	–	–	–	–	–	PVS1 + PS2 + PM2	Pathogenic
Case 4	c.6340-1G > A	*De novo*	–	–	–	–	–	–	–	PVS1 + PS2 + PM2	Pathogenic
Case 5	p.Arg68Leu	*De novo*	–	–	0.002 (D)	0.319 (B)	0.999 (D)	0.753 (D)	–0.73	PS2 + PM2 + PP3	Likely pathogenic
Case 6	p.Gly1322Glu	*De novo*	–	–	0.011 (D)	1 (D)	1 (D)	0.687 (D)	–1.47	PS2 + PM2 + PP3	Likely pathogenic
Case 7	p.Arg1678Cys	*De novo*	–	–	0 (D)	1 (D)	1 (D)	0.833 (D)	–0.89	PS2 + PM2 + PP3	Likely pathogenic
Case 8	p.Ser1798Leu	*De novo*	–	–	0 (D)	0.998 (D)	1 (D)	0.794 (D)	–0.15	PS2 + PM2 + PP3	Likely pathogenic
Case 9	p.Ser1078Leu p.Glu2021Lys	Paternal Maternal	1.4 × 10^–5^ 3.7 × 10^–4^	2.1 × 10^–4^ 4.8 × 10^–3^	0.09 (T) 0.072 (T)	0.057 (B) 0.441 (B)	1 (P) 1 (D)	0.619 (D) 0.25 (D)	0.44 0.28	PM2 + PP3 PM2 + PP3	Uncertain significance
Case 10	p.Pro1993Leu p.Ile1631Val	Paternal Maternal	6.1 × 10^–6^ 1.3 × 10^–4^	– 1.9 × 10^–3^	0.304 (T) 0.58 (T)	0.738 (D) 0.262 (B)	1 (D) 0.997 (D)	0.221 (D) 0.076 (D)	0.19 –1.17	PM2 + PP3 PM2 + PP3	Uncertain significance

*ACMG, American College of Medical Genetics and Genomics; B, benign; D, damaging; DDG, protein stability indicated by free energy change value; MAF, minor allele frequency from gnomAD; MAF-EAS, minor allele frequency from gnomAD-East Asian population; M_CAP, Mendelian Clinically Applicable Pathogenicity; P, polymorphism; PM2, absent in population databases; PP2_Var, Polyphen2_HVAR; PP3, multiple lines of computational evidence support a deleterious effect on the gene/gene product; PS2, De novo (paternity and maternity confirmed); PVS1, predicted null variant in a gene where loss of function (LOF) is a known mechanism of disease; SIFT, Sorting Intolerant From Tolerant; T, tolerable.*

*^a^Typical results of damage effect prediction of the CACNA1A mutations in this table were selected from 23 algorithms in silico missense prediction (http://varcards.biols.ac.cn/).*

*means a premature termination of the protein caused by a frameshift mutation.

The c.2963_2964insG/p.Gly989Argfs*78 mutation was considered to potentially pathogenic by yielding a truncated transcript that gave rise to a non-functional Cav2.1 protein or haploinsufficiency. The three canonical splice site mutations (c.3089 + 1G > A, c.4755 + 1G > T, and c.6340-1G > A) could destroy the original splice donor or acceptor site that generally resulted in the skipping of the single exon or multiexon with consequent translational frameshift. All of the eight missense mutations were predicted to be damaging by at least one of the commonly used *in silico* prediction tools (Table 2). The amino acid sequence alignment indicated that Arg68Leu, Gly1322Glu, Arg1678Cys, Ser1798Leu, Pro1993Leu, and Glu2021Lys were located at residues that are highly conserved in various species; Ile1631Val are highly conserved in vertebrates but less so in lower animals (Figure 2B). The Ser1078Leu was located at a less conserved site but was predicted to be conserved by GERP (score = 5.17), phyloP (score = 5.429), and SiPhy (score = 17.443). Furthermore, I-Mutant 3.0 program showed that Arg68Leu, Gly1322Glu, Ile1631Val, and Arg1678Cys mutants have a strong influence on protein stability (Table 2).

None of the 10 patients had pathogenic or likely pathogenic mutations in the genes known to be associated with epileptic phenotypes (Wang et al., 2017) except *CACNA1A* mutations.

### Clinical Features

**FIGURE 3:**
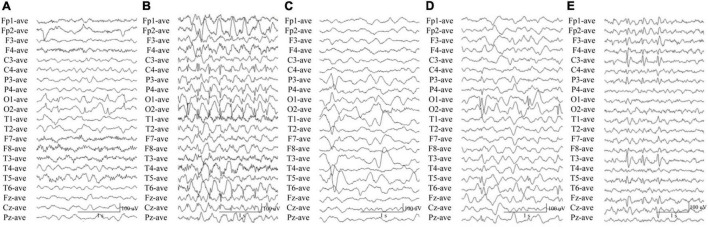
Electroencephalography data of the cases with *CACNA1A* mutations. **(A)** Interictal EEG in case 2 showed bilateral occipital spike and slow waves. **(B)** Interictal EEG in case 4 showed generalized 3 Hz spike and slow waves. **(C)** Interictal EEG in case 6 showed left parietal and temporal sharp waves and focal spike and slow waves. **(D)** Interictal EEG in case 8 showed bilateral occipital spikes and focal spike and slow waves. **(E)** Interictal EEG in case 10 showed left parietal and temporal spikes.

In this study, we identified *CACNA1A* mutations in 10 unrelated cases. The seizure onset age of the ten cases ranged from 3 months to 11 years old, with a median age of onset of 3.5 years. Eight of the cases were diagnosed as partial epilepsy, including six cases with *de novo* missense/null mutations and two with compound heterozygous missense mutations. They had simple/complex partial seizures or secondarily generalized tonic-clonic seizures. They all had focal epileptic discharges with normal backgrounds or trends of generalization especially during sleep (Figures 3A,C–E). The remaining two cases were diagnosed as childhood absence epilepsy and carried two *de novo* canonical splice site mutations. They experienced frequent absence seizures and detected ictal or interictal generalized 3 HZ spike and slow waves on EEG recordings (Figure 3B). A patient (case 2) also carried a canonical splice site mutation (c.3089 + 1G > A) and was diagnosed as partial epilepsy, her EEGs presented bilateral occipital epileptic discharges with features of idiopathic epilepsies (Figure 3A). These findings indicated that splice site mutations of *CACNA1A* were potentially associated with generalized epilepsies or idiopathic epilepsies. All of the ten cases were ultimately seizure-free after antiepileptic treatment, although frequent epileptic seizures were observed in four cases (Cases 3, 4, 6, and 9; Table 1). One patient (Case 3) had antecedent febrile seizures. Three patients had mild intellectual abnormalities (Cases 5, 6, and 8; Table 1). All the ten cases were born by normal delivery, and the brain MRI findings were normal. No ataxia or migraine were observed in any of them.

### Genotype-Phenotype Correlation

**FIGURE 4:**
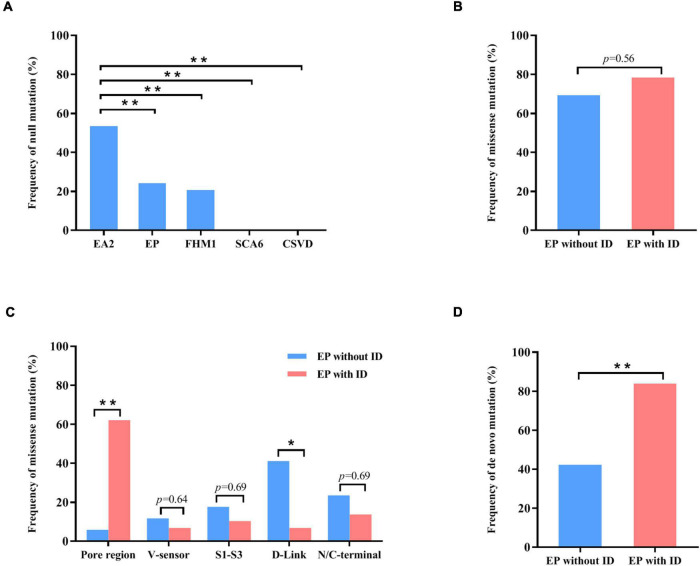
Genotype–phenotype correlations of *CACNA1A* mutations. **(A)** The frequency of null mutations in *CACNA1A* for each phenotype. The values are expressed as the percentage of cases with null mutations (cases with null mutations/total cases) in each group. **(B)** The frequency of null mutations in *CACNA1A* for epilepsy without intellectual disability and epilepsy with intellectual disability. **(C)** The frequency of missense mutants in various regions of the Cav2.1 channel for epilepsy and epilepsy with intellectual disability. **(D)** The frequency of de novo mutations in *CACNA1A* for epilepsy and epilepsy with intellectual disability. Fisher’s exact test was used for statistical analysis of the differences between each group. CSVD, cerebral small vessel disease (*n* = 18). EA2, episodic ataxia 2 (*n* = 155). EP, epilepsy (*n* = 63). EP without ID, epilepsy without intellectual disability (*n* = 26). EP with ID, epilepsy with intellectual disability (*n* = 37). FHM1, familial hemiplegic migraine 1 (*n* = 53). SCA6, spinocerebellar ataxia 6 (*n* = 7). * *P* < 0.05; ** *P* < 0.01.

To explore the correlation between genotype and phenotype, we systematically reviewed all reported *CACNA1A* mutations. Previously, 312 mutations have been reported, including 115 null mutations, 183 missense mutations, 10 in-frame insertion/deletion mutations, and 4 (CAG)n dynamic mutations. These mutations were associated with a variety of clinical phenotypes that included EA2, FHM1, SCA6, CSVD (cerebral small vessel disease), and epilepsies. EA2 group present a significantly higher frequency of null mutation than the groups of epilepsy (*P* = 7.92 × 10^–5^), FHM1 (*P* = 2.85 × 10^–5^), SCA6 (*P* = 5.69 × 10^–3^), or CSVD (*P* = 3.77 × 10^–6^) (Figure 4A).

Cav2.1 encoded by *CACNA1A*, contains four homologous domains (DI-DIV) with six helical transmembrane segments (S1–S6). The S4 segments of each repeat serve as actual voltage sensors while S5 and S6 segments together with S5-S6 loop of each repeat form the channel pore (Figure 2A; Striessnig, 2021). In the present study, *de novo* missense mutations, except the Arg68Leu, were all located at pore region or near the voltage sensor region while compound heterozygous missense mutations were mainly located at linker region or C-terminal. We analyzed the data together with that from literature (Figure 2B). Previous studies have shown *CACNA1A* mutations potentially have an association with developmental abnormalities (Allen et al., 2013; Damaj et al., 2015; Epi4k Consortium, 2016). In this cohort, three cases of partial epilepsy also have ID. We then analyzed the epilepsies with ID and those without ID (Supplementary Table 1). No statistical difference in the frequency of missense was observed between the two epilepsy sub-groups (Figure 4B). However, it was found that missense mutations in the epilepsy with ID were more frequently located in the pore region than those in the epilepsy without ID (*P* = 1.67 × 10^–4^) (Figure 4C), suggesting a molecular sub-region effect. Moreover, the cases in the epilepsy with ID group had a higher percentage of *de novo* mutations than those in the epilepsy without ID (*P* = 1.92 × 10^–3^) (Figure 4D), suggesting a potential correlation between epileptic phenotype severity and mutation origins.

## Discussion

Previous studies have showed that the clinical phenotypes caused by *CACNA1A* mutations comprises a huge group of phenotypic heterogeneity, such as EA2, FHM1, SCA6, and DEE42 that was a severe form of epilepsy (Ophoff et al., 1996; Jodice et al., 1997; Zhuchenko et al., 1997; Terwindt et al., 2002; Jen et al., 2007; Rajakulendran et al., 2012; Allen et al., 2013; Epi4k Consortium, 2016). In the present study, we identified 12 *CACNA1A* mutations in ten cases of mild form of epilepsy, including four *de novo* null mutations, four *de novo* missense mutations, and two pairs of compound heterozygous missense mutations. The eight *de novo* mutations were evaluated as pathogenic or likely pathogenic mutations according to the criteria of ACMG (Table 2). Although both of the compound heterozygous missense mutations were evaluated as uncertain significance, the frequencies of the recessive *CACNA1A* mutations identified in this cohort were significantly higher than that in the controls of East Asian and all populations. This study suggested that *CACNA1A* gene is potentially associated with epilepsy. The patients with *CACNA1A* mutations may present epilepsy without ataxia or migraine. The spectrum of epileptic phenotypes potentially ranged from the mild form of epilepsies such as absence epilepsy or partial epilepsy, to the severe form of developmental epileptic encephalopathy.

The *CACNA1A* gene is predominantly expressed in neuron and plays a critical role in membrane excitability and neurotransmission release (Diriong et al., 1995; Kramer et al., 1995; Teh et al., 1995). *Cacna1a* knockout mouse model exhibited ataxia and epilepsy seizures.^6^ The clinical phenotypes caused by *CACNA1A* mutations were highly concordant with that of *Cacna1a* knockout mouse model. Thus, *CACNA1A* loss of function may be the potentially pathogenic mechanism. *CACNA1A* mutations identified in this study included four null mutations and two compound heterozygous mutations that were potentially associated with a loss of function. The remaining four *de novo* missense mutations with protein substitution were located at the most highly conserved residue in the protein sequence alignments. The Gly1322Glu, Arg1678Cys, and Ser1798Leu mutants were located at pore region or near the voltage sensor region. The Arg68Leu and Gly1322Glu mutants have a strong influence on protein stability (Table 2). Therefore, the four *de novo* missense mutations were also considered to be potentially deleterious because of the possibility of giving rise to alteration of the structure of pore region/voltage sensor region or influencing the protein stability. However, the accurate functional consequence of the newly identified missense mutations was unknown. Previous studies have shown that *SCN1A* missense mutations in the pore region were characterized by loss of function (Meng et al., 2015). Currently, data on functional alteration of *CACNA1A* mutations is limited and did not permit a conclusion. Functional alteration of other type, such as gain of function, could not be excluded. Hence, the correlation between functional consequence and location of *CACNA1A* mutations warrants further studies.

Cav2.1 encoded by *CACNA1A*, is the pore-forming alpha-1A subunit of VGCC and contains four homologous domains (DI-DIV) with six helical transmembrane segments (S1–S6) (Figure 2A; Striessnig, 2021). Previously, *CACNA1A* have been established an association with DEE42 (Allen et al., 2013; Epi4k Consortium, 2016). In the present study, we identified *CACNA1A* mutations in the cases with relatively mild epilepsies. Most of the mutations were null mutations or in the pore-regions that would cause loss of function. The four missense mutations constituted two pairs of compound heterozygous mutations that were located at linker region or C-terminal. While single heterozygous variant was not pathogenic, the compound heterozygous mutations became potentially pathogenic. Our further analyses showed that missense mutations in the epilepsy with ID were more frequently located in the pore region than those in the epilepsy without ID. These findings potentially suggested a molecular sub-region effect. This was also supported by a recent study that showed missense mutations located in the pore region were associated with severe epileptic encephalopathy, in spite of the difference in functional alteration (Jiang et al., 2019). Besides, two of splice site mutations were associated with generalized epilepsies characterized by absence seizures, suggesting a possible genotype-phenotype association that warrants further verification.

Previously, *CACNA1A* mutations were mainly associated with paroxysmal diseases such as EA2. The Ser1798Leu mutation identified in this study (case 8 with epilepsy) has been previously reported in a case of episodic ataxia 2 (EA2) (Ohba et al., 2013). Experiments in animals showed that *Cacna1a* knockout caused ataxia and epilepsy seizures (Pietrobon, 2005). The present study demonstrated that EA2 was more frequently associated with null mutations than epilepsy. However, it is unknown why the same mutation was associated with different phenotypes. Previous studies in *Cacna1a* knockout mice have indicated that loss of Cav2.1 channel would probably affect the function of other voltage-gated calcium channels (Reinson et al., 2016), which added one more factor on the expression of phenotype. Other mechanisms, such as genetic background and interactive genes, should be studied further.

In summary, we identified *CACNA1A* mutations in ten unrelated cases with relatively mild and pure epilepsy. All patients had favorable outcome with antiepileptic treatment without ataxia or migraine. Further analysis showed the clinical phenotypes variability is potentially associated with mutation type, molecular sub-regional effect, and inheritance pattern, which would help understanding the mechanism underlying phenotypical heterogeneity.

## Data Availability Statement

The datasets presented in this study can be found in online repositories. The names of the repository/repositories and accession number(s) can be found in the article/Supplementary Material.

## Ethics Statement

The studies involving human participants were reviewed and approved by Ethics Committee of the Second Affiliated Hospital of Guangzhou Medical University. Written informed consent to participate in this study was provided by the participants’ legal guardian/next of kin. Written informed consent was obtained from the individual(s), and minor(s)’ legal guardian/next of kin, for the publication of any potentially identifiable images or data included in this article.

## Author Contributions

X-LL and JW designed the study. Z-JL, X-YL, D-TL, C-FC, MJ, BL, NH, B-ML, W-JB, X-QT, HL, and Y-HY completed the recruitment of the patients and the analysis of the clinical data. L-DG, Y-WS, and JW completed the analysis of the genetic data. C-FC and Z-JL prepared the figures. X-LL and JW wrote the manuscript. Y-HY revised the manuscript. All authors have read and approved the final manuscript.

## Conflict of Interest

The authors declare that the research was conducted in the absence of any commercial or financial relationships that could be construed as a potential conflict of interest.

## Publisher’s Note

All claims expressed in this article are solely those of the authors and do not necessarily represent those of their affiliated organizations, or those of the publisher, the editors and the reviewers. Any product that may be evaluated in this article, or claim that may be made by its manufacturer, is not guaranteed or endorsed by the publisher.

## Footnotes

1
http://gnomad.broadinstitute.org/

2
http://varcards.biols.ac.cn/

3
http://gpcr2.biocomp.unibo.it/cgi/predictors/I-Mutant3.0/I-Mutant3.0.cgi

4
http://www.hgmd.cf.ac.uk/ac/index.php

5
http://www.ncbi.nlm.nih.gov/pubmed/

6
http://www.informatics.jax.org/marker/MGI:109482
