# Supplementary material for: CACNA1A Mutations Associated With Epilepsies and Their Molecular Sub-Regional Implications
Source: Front Mol Neurosci. 2022 May 4;15:860662. doi: 10.3389/fnmol.2022.860662 (PMC9116572; doi:10.3389/fnmol.2022.860662)
Supplement: Supplementary file 1 [file Table_1.DOCX]

**Supplementary Data**

**Table S1. Epilepsy-related *CACNA1A* mutations and their** **locations of genic sub-region**

| Mutation type | Nucleotide change | Amino acid change | Inheritance | Location | Phenotype | Reference | |
| --- | --- | --- | --- | --- | --- | --- | --- |
| Cases of epilepsy without intellectual disability | | | | | | |  |
| Missense | c.301G>C | p.Glu101Gln | De novo | DIS1 | Epilepsy of infancy with migrating focal seizures | (Epi4K Consortium, 2016) | |
|  | c.410A>G | p.Glu137Gly | De novo | DIS2 | Absence epilepsy with ataxia | (Du et al., 2017) | |
|  | c.677T>G | p.Leu226Trp | Maternal | DIS4-S5 | juvenile myoclonic epilepsy | (Alehabib et al., 2021) | |
|  | c.1242G>T | p.Arg414Ser | Unknown | DI-DII | Epilepsy, early-onset | (Demos et al., 2019) | |
|  | c.1430G>A | p.Arg477His | Unknown | DI-DII | Epilepsy, idiopathic | (Klassen et al., 2011) | |
|  | c.2195A>C | p.Glu732Ala | Unknown | DII-DIII | Juvenile myoclonic epilepsy | (Lee et al., 2018) | |
|  | c.2663A>T | p.Gln888Leu | De novo | DII-DIII | Epilepsy, early-onset | (Staněk et al., 2018) | |
|  | c.3233C>T | p.Ser1078Leu | Paternal | DII-DIII | Partial epilepsy | This study | |
|  | c.3239C>A | p.Ala1080Asp | Unknown | DII-DIII | Juvenile myoclonic epilepsy | (Lee et al., 2018) | |
|  | c.3313G>A | p.Gly1105Ser | Unknown | DII-DIII | Epilepsy, idiopathic | (Klassen et al., 2011) | |
|  | c.4891A>G | p.Ile1631Val | Maternal | DIVS3 | Partial epilepsy | This study | |
|  | c.5017C>T | p.Arg1673Cys | Unknown | DIVS4-S5 | Generalised convulsive epilepsy & hemiplegia | (Butler et al., 2017) | |
|  | c.5044C>T | p.Arg1678Cys | De novo | DIVS4-S5 | Partial epilepsy | This study | |
|  | c.5263G>A | p.Gly1755Arg | De novo | DIVS5-S6 | Epilepsy with typical absence seizures | (Helbig et al., 2016) | |
|  | c.5442T>G | p.Phe1814Leu | De novo | C-terminal | Epilepsy | (Zhang et al., 2020) | |
|  | c.5900G>A | p.Arg1967Gln | Unknown | C-terminal | Epilepsy, idiopathic | (Klassen et al., 2011) | |
|  | c.5978C>T | p.Pro1993Leu | Paternal | C-terminal | Partial epilepsy | This study | |
|  | c.6061G>A | p.Glu2021Lys | Maternal | C-terminal | Partial epilepsy | This study | |
| Null | c.714delT | p.Ile239Phefs*5 | Unknown | DIS5 | Juvenile myoclonic epilepsy | (Lee et al., 2018) | |
|  | c.1915C>T | p.Gln639* | De novo | DIIS5-S6 | Epilepsy, early-onset | (Helbig et al., 2016) | |
|  | c.2324G>A | p.Trp775* | Unknown | DII-DIII | Epilepsy, childhood-onset | (Symonds et al., 2019) | |
|  | c.2975dupG | p.Gly989Argfs*78 | De novo | DII-DIII | Partial epilepsy | This study | |
|  | c.3089+1G>A | – | De novo | DII-DIII | Partial epilepsy | This study | |
|  | c.4189C>T | p.Gln1397* | Unknown | DIIIS5-S6 | Epilepsy, idiopathic | (Klassen et al., 2011) | |
|  | c.4755+1G>T | – | De novo | DIVS1 | Childhood absence epilepsy | This study | |
|  | c.6340-1G>A | – | De novo | C-terminal | Childhood absence epilepsy | This study | |
| Cases of epilepsy with intellectual disability | | | | | | |  |
| Missense | c.165A＞C | p.Arg55Ser | De novo | N-terminal | Epilepsy and developmental delay | (Niu et al., 2022) | |
|  | c.185A＞G | p.Tyr62Cys | De novo | N-terminal | Epilepsy and developmental delay | (Niu et al., 2022) | |
|  | c.203G>T | p.Arg68Leu | De novo | N-terminal | Partial epilepsy and intellectual disability | This study | |
|  | c.316G>A | p.Ala106Thr | Inherited | DIS1 | Epileptic encephalopathy | (Zhu et al., 2017) | |
|  | c.689G>T | p.Gly230Val | De novo | DIS5 | Developmental and epileptic encephalopathy | (Jiang et al., 2019) | |
|  | c.692T>G | p.Leu231Arg | De novo | DIS5 | Status epilepticus | (Wang et al., 2021) | |
|  | c.848A>G | p.Asn283Ser | De novo | DIS5-S6 | Epilepsy and developmental delay | (Niu et al., 2022) | |
|  | c.889G＞A | p.Gly297Arg | De novo | DIS5-S6 | Epilepsy and developmental delay | (Tantsis et al., 2016) | |
|  | c.997A>G | p.Asn333Asp | Unknown | DIS5-S6 | Epileptic encephalopathy, infantile, type 42 | (Nair et al., 2018) | |
|  | c.1886C>T | p.Ala629Val | De novo | DIIS5 | Epilepsy and intellectual disability | (Benson et al., 2020) | |
|  | c.2122G>A | p.Val708Met | De novo | DIIS6 | Epileptic encephalopathy | (Hirasawa-Inoue et al., 2019) | |
|  | c.2131G>A | p.Ala711Thr | De novo | DIIS6 | Rett syndrome, atypical | (Epperson et al., 2018) | |
|  | c.2134A>G | p.Ile712Val | De novo | DIIS6 | Episodic seizures, ataxia, and migraine with motor regression | (Guerin et al., 2008) | |
|  | c.2137G>A | p.Ala713Thr | De novo | DIIS6 | Lennox-Gastaut syndrome | (Allen et al., 2013) | |
|  | c.2276T＞C | p.Ile759Thr | Paternal | DII-DIII | Epilepsy and developmental delay | (Niu et al., 2022) | |
|  | c.3965G>A | p.Gly1322Glu | De novo | DIIIS3 | Partial epilepsy and intellectual disability | This study | |
|  | c.3968G＞A | p.Gly1323Glu | De novo | DIIIS3 | Epilepsy and developmental delay | (Niu et al., 2022) | |
|  | c.4070T>G | p.Ile1357Ser | De novo | DIIIS4 | Developmental and epileptic encephalopathy | (Jiang et al., 2019) | |
|  | c.4118C>T | p.Ser1373Leu | De novo | DIIIS4-S5 | Encephalopathy, epileptic | (Byers et al., 2016) | |
|  | c.4129G>T | p.Val1377Phe | De novo | DIIIS5 | Status epilepticus | (Wang et al., 2021) | |
|  | c.4177G＞A | p.Val1393Met | De novo | DIIIS5 | Epilepsy and developmental delay | (Butler et al., 2017) | |
|  | c.4186G>A | p.V1396M | De novo | DIIIS5 | Developmental and epileptic encephalopathy | (Travaglini et al., 2017) | |
|  | c.4306T>A | p.Trp1436Arg | Maternal | DIIIS5-S6 | Epileptic encephalopathy, early onset with progressive cerebellar & optic nerve atrophy | (Reinson et al., 2016) | |
|  | c.4406C＞T | p.Ser1469Leu | De novo | DIIIS5-S6 | Epilepsy and developmental delay | (Niu et al., 2022) | |
|  | c.4522G>T | p.Ala1508Ser | De novo | DIIIS6 | Epileptic encephalopathy, early onset | (Epi4K Consortium, 2016) | |
|  | c.4552G>A | p.Gly1518Arg | De novo | DIII-DIV | Epileptic encephalopathy, early infantile | (Retterer et al., 2016) | |
|  | c.5075T>A | p.Leu1692Gln | De novo | DIVS5 | Early ischemic stroke, intractable epilepsy and global developmental delay | (Gudenkauf et al., 2020) | |
|  | c.5393C>T | p.Ser1798Leu | De novo | DIVS6 | Partial epilepsy and intellectual disability | This study | |
|  | c.5422G>A | p.Ala1808Thr | De novo | C-terminal | Status epilepticus | (Wang et al., 2021) | |
| Null | c.472_478del | p.Ala158Thrfs*6 | Paternal | DIS2-S3 | Epileptic encephalopathy, early onset with progressive cerebellar & optic nerve atrophy | (Reinson et al., 2016) | |
|  | c.506G＞A | p.Trp169* | De novo | DIS3 | Epilepsy and developmental delay | (Niu et al., 2022) | |
|  | c.2053C＞T | p.Gln685* | De novo | DIIS5-S6 | Epilepsy and developmental delay | (Niu et al., 2022) | |
|  | c.3825+1G>A | – | De novo | DIIIS1-S2 | Epileptic encephalopathy, infantile, type 42 | (Ziats et al., 2020) | |
|  | c.2039_2040del | p.Gln680Argfs*100 | De novo | DII-DIII | Epilepsy and developmental delay | (Niu et al., 2022) | |
|  | c.6530-1G＞C | – | Maternal | C-terminal | Epilepsy and developmental delay | (Niu et al., 2022) | |
|  | 0.7Mb deletion incl entire gene | – | De novo | – | Mental retardation & epilepsy with infantile spasms | (Auvin et al., 2009) | |
| Inframe | c.4070_4072del | p.Ile1357del | De novo | DIIIS4 | Epilepsy and autism spectrum disorder | (Long et al., 2019) | |

**References**

Alehabib, E., Kokotović, T., Ranji-Burachaloo, S., Tafakhori, A., Ramshe, S.M., Esmaeilizadeh, Z., et al. (2021). Leu226Trp CACNA1A variant associated with juvenile myoclonic epilepsy with and without intellectual disability. *Clin Neurol Neurosurg* 213**,** 107108. doi: 10.1016/j.clineuro.2021.107108.

Allen, A.S., Berkovic, S.F., Cossette, P., Delanty, N., Dlugos, D., Eichler, E.E., et al. (2013). De novo mutations in epileptic encephalopathies. *Nature* 501(7466)**,** 217-221.

Auvin, S., Holder-Espinasse, M., Lamblin, M.D., and Andrieux, J. (2009). Array-CGH detection of a de novo 0.7-Mb deletion in 19p13.13 including CACNA1A associated with mental retardation and epilepsy with infantile spasms. *Epilepsia* 50(11)**,** 2501-2503. doi: 10.1111/j.1528-1167.2009.02189.x.

Benson, K.A., White, M., Allen, N.M., Byrne, S., Carton, R., Comerford, E., et al. (2020). A comparison of genomic diagnostics in adults and children with epilepsy and comorbid intellectual disability. *Eur J Hum Genet* 28(8)**,** 1066-1077.

Butler, K.M., da Silva, C., Alexander, J.J., Hegde, M., and Escayg, A. (2017). Diagnostic Yield From 339 Epilepsy Patients Screened on a Clinical Gene Panel. *Pediatr Neurol* 77**,** 61-66.

Byers, H.M., Beatty, C.W., Hahn, S.H., and Gospe, S.M., Jr. (2016). Dramatic Response After Lamotrigine in a Patient With Epileptic Encephalopathy and a De NovoCACNA1A Variant. *Pediatr Neurol* 60**,** 79-82.

Demos, M., Guella, I., DeGuzman, C., McKenzie, M.B., Buerki, S.E., Evans, D.M., et al. (2019). Diagnostic Yield and Treatment Impact of Targeted Exome Sequencing in Early-Onset Epilepsy. *Front Neurol* 10**,** 434.

Du, X., Chen, Y., Zhao, Y., Luo, W., Cen, Z., and Hao, W. (2017). Dramatic response to pyridoxine in a girl with absence epilepsy with ataxia caused by a de novo CACNA1A mutation. *Seizure* 45**,** 189-191. doi: 10.1016/j.seizure.2016.12.020.

Epi4K Consortium (2016). De Novo Mutations in SLC1A2 and CACNA1A Are Important Causes of Epileptic Encephalopathies. *Am J Hum Genet* 99(2)**,** 287-298.

Epperson, M.V., Haws, M.E., Standridge, S.M., and Gilbert, D.L. (2018). An Atypical Rett Syndrome Phenotype Due to a Novel Missense Mutation in CACNA1A. *J Child Neurol* 33(4)**,** 286-289.

Gudenkauf, F.J., Azamian, M.S., Hunter, J.V., Nayak, A., and Lalani, S.R. (2020). A novel CACNA1A variant in a child with early stroke and intractable epilepsy. *Mol Genet Genomic Med* 8(10)**,** e1383.

Guerin, A.A., Feigenbaum, A., Donner, E.J., and Yoon, G. (2008). Stepwise developmental regression associated with novel CACNA1A mutation. *Pediatr Neurol* 39(5)**,** 363-364. doi: 10.1016/j.pediatrneurol.2008.07.030.

Helbig, K.L., Farwell Hagman, K.D., Shinde, D.N., Mroske, C., Powis, Z., Li, S., et al. (2016). Diagnostic exome sequencing provides a molecular diagnosis for a significant proportion of patients with epilepsy. *Genet Med* 18(9)**,** 898-905. doi: 10.1038/gim.2015.186.

Hirasawa-Inoue, A., Ishiyama, A., Takeshita, E., Shimizu-Motohashi, Y., Saito, T., Komaki, H., et al. (2019). Single-fiber electromyography-based diagnosis of CACNA1A mutation in children: A potential role of the electrodiagnosis in the era of whole exome sequencing. *Brain Dev* 41(10)**,** 905-909. doi: 10.1016/j.braindev.2019.06.006.

Jiang, X., Raju, P.K., D'Avanzo, N., Lachance, M., Pepin, J., Dubeau, F., et al. (2019). Both gain-of-function and loss-of-function de novo CACNA1A mutations cause severe developmental epileptic encephalopathies in the spectrum of Lennox-Gastaut syndrome. *Epilepsia* 60(9)**,** 1881-1894. doi: 10.1111/epi.16316.

Klassen, T., Davis, C., Goldman, A., Burgess, D., Chen, T., Wheeler, D., et al. (2011). Exome sequencing of ion channel genes reveals complex profiles confounding personal risk assessment in epilepsy. *Cell* 145(7)**,** 1036-1048.

Lee, C.G., Lee, J., and Lee, M. (2018). Multi-gene panel testing in Korean patients with common genetic generalized epilepsy syndromes. *PLoS One* 13(6)**,** e0199321.

Long, S., Zhou, H., Li, S., Wang, T., Ma, Y., Li, C., et al. (2019). The Clinical and Genetic Features of Co-occurring Epilepsy and Autism Spectrum Disorder in Chinese Children. *Front Neurol* 10**,** 505.

Nair, P., Sabbagh, S., Mansour, H., Fawaz, A., Hmaimess, G., Noun, P., et al. (2018). Contribution of next generation sequencing in pediatric practice in Lebanon. A Study on 213 cases. *Mol Genet Genomic Med* 6(6)**,** 1041-1052.

Niu, X., Yang, Y., Chen, Y., Cheng, M., Liu, M., Ding, C., et al. (2022). Genotype-phenotype correlation of CACNA1A variants in children with epilepsy. *Dev Med Child Neurol* 64(1)**,** 105-111. doi: 10.1111/dmcn.14985.

Reinson, K., Õiglane-Shlik, E., Talvik, I., Vaher, U., Õunapuu, A., Ennok, M., et al. (2016). Biallelic CACNA1A mutations cause early onset epileptic encephalopathy with progressive cerebral, cerebellar, and optic nerve atrophy. *Am J Med Genet A* 170(8)**,** 2173-2176. doi: 10.1002/ajmg.a.37678.

Retterer, K., Juusola, J., Cho, M.T., Vitazka, P., Millan, F., Gibellini, F., et al. (2016). Clinical application of whole-exome sequencing across clinical indications. *Genet Med* 18(7)**,** 696-704. doi: 10.1038/gim.2015.148.

Staněk, D., Laššuthová, P., Štěrbová, K., Vlčková, M., Neupauerová, J., Krůtová, M., et al. (2018). Detection rate of causal variants in severe childhood epilepsy is highest in patients with seizure onset within the first four weeks of life. *Orphanet J Rare Dis* 13(1)**,** 71.

Symonds, J.D., Zuberi, S.M., Stewart, K., McLellan, A., O'Regan, M., MacLeod, S., et al. (2019). Incidence and phenotypes of childhood-onset genetic epilepsies: a prospective population-based national cohort. *Brain* 142(8)**,** 2303-2318.

Tantsis, E.M., Gill, D., Griffiths, L., Gupta, S., Lawson, J., Maksemous, N., et al. (2016). Eye movement disorders are an early manifestation of CACNA1A mutations in children. *Dev Med Child Neurol* 58(6)**,** 639-644. doi: 10.1111/dmcn.13033.

Travaglini, L., Nardella, M., Bellacchio, E., D'Amico, A., Capuano, A., Frusciante, R., et al. (2017). Missense mutations of CACNA1A are a frequent cause of autosomal dominant nonprogressive congenital ataxia. *Eur J Paediatr Neurol* 21(3)**,** 450-456. doi: 10.1016/j.ejpn.2016.11.005.

Wang, T., Wang, J., Ma, Y., Zhou, H., Ding, D., Li, C., et al. (2021). High genetic burden in 163 Chinese children with status epilepticus. *Seizure* 84**,** 40-46. doi: 10.1016/j.seizure.2020.10.032.

Zhang, L., Wen, Y., Zhang, Q., Chen, Y., Wang, J., Shi, K., et al. (2020). CACNA1A Gene Variants in Eight Chinese Patients With a Wide Range of Phenotypes. *Front Pediatr* 8**,** 577544.

Zhu, X., Padmanabhan, R., Copeland, B., Bridgers, J., Ren, Z., Kamalakaran, S., et al. (2017). A case-control collapsing analysis identifies epilepsy genes implicated in trio sequencing studies focused on de novo mutations. *PLoS Genet* 13(11)**,** e1007104.

Ziats, M.N., Ahmad, A., Bernat, J.A., Fisher, R., Glassford, M., Hannibal, M.C., et al. (2020). Genotype-phenotype analysis of 523 patients by genetics evaluation and clinical exome sequencing. *Pediatr Res* 87(4)**,** 735-739.
